# Ailanthoidol, a Neolignan, Suppresses TGF-β1-Induced HepG2 Hepatoblastoma Cell Progression

**DOI:** 10.3390/biomedicines9091110

**Published:** 2021-08-30

**Authors:** Tsui-Hwa Tseng, Huei-Jane Lee, Yean-Jang Lee, Ko-Chao Lee, Chien-Heng Shen, Hsing-Chun Kuo

**Affiliations:** 1 Department of Medical Applied Chemistry, Chung Shan Medical University, Taichung 40201, Taiwan; tht@csmu.edu.tw; 2 Department of Medical Education, Chung Shan Medical University Hospital, Taichung 40201, Taiwan; 3 Department of Biochemistry, School of Medicine, College of Medicine, Chung Shan Medical University, Taichung 40201, Taiwan; lhj@csmu.edu.tw; 4 Department of Chemistry, National Changhua University of Education, Changhua 50007, Taiwan; leeyj@cc.ncue.edu.tw; 5 Division of Colorectal Surgery, Department of Surgery, Chang Gung Memorial Hospital, Kaohsiung Medical Center, Chang Gung University College of Medicine, Kaohsiung 83301, Taiwan; kmch4329@gmail.com; 6 Department of Hepato-Gastroenterology, Chang Gung Memorial Hospital, Chiayi 61363, Taiwan; 7 Department of Nursing, Division of Basic Medical Sciences, Chang Gung University of Science and Technology, Chiayi 61363, Taiwan; 8 Research Fellow, Chang Gung Memorial Hospital, Chiayi 61363, Taiwan; 9 Research Center for Food and Cosmetic Safety, College of Human Ecology, Chang Gung University of Science and Technology, Taoyuan 33303, Taiwan; 10 Chronic Diseases and Health Promotion Research Center, Chang Gung University of Science and Technology, Chiayi 61363, Taiwan

**Keywords:** Ailanthoidol, TGF-β1, p-38MAPK, anti-hepatic cancer progression

## Abstract

Ailanthoidol (ATD), a neolignan, possessed an antitumor promotion effect in the mouse skin model in our previous investigation. However, other antitumor properties remain to be elucidated. Liver cancer is a major cause of death in the world, and its prognosis and survival rate are poor. Therefore, the prevention and therapy of liver cancer have received much attention. TGF (transforming growth factor)-β1, a cytokine, plays a critical role in the progression of liver cancer. This study determined the inhibitory effects of ATD on the migration and invasion induced by TGF-β1 in HepG2 hepatoblastoma cells. Furthermore, ATD reduced the TGF-β1-promoted colony number of HepG2 hepatoblastoma cells. In addition to reversing TGF-β1-induced cell scattering, ATD suppressed TGF-β1-induced expression of integrin α3, vimentin, N-cadherin, and matrix metalloproteinase 2 (MMP2). Finally, this study found that ATD significantly inhibited TGF-β1-promoted phosphorylation of p-38 mitogen-activated protein kinase (MAPK) and Smad 2. Furthermore, the administration of SB203580 (p38MAPK inhibitor) suppressed TGF-β1-induced expression of integrin α3, N-cadherin, and MMP2. These results demonstrate a novel mechanism of ATD against progression of liver cancer.

## 1. Introduction

Liver cancer is a common global malignancy with a high recurrence rate and poor prognosis [1]. Metastasis has been notably reported as the major cause of treatment failure in patients with liver cancer [2]. Metastasis, which is a multistep process, includes migration, invasion, implantation, colonization, and proliferation [3]. Migration and invasion of the primary tumor cells occur through the basement membrane and extracellular matrix, and form secondary tumors at distant sites, which are mediated by integrins and proteases. Recently, it has been suggested that epithelial to mesenchymal transition (EMT) contributes to the early spreading of cancer cells [4]. During EMT epithelial, polarized cells become motile mesenchymal appearing cells, where cell–cell attachment is lost, epithelial markers are downregulated, and mesenchymal markers are upregulated [5]; thus, the search for an agent to block metastasis in liver cancer is a hope to sustain life.

Chronic inflammation is associated with the high incidence rate of several cancers, including liver cancer [6]. The inflammatory cells produce a vast number of cytokines, growth factors, prostaglandin, and chemokines, which may contribute to hepatic tumorigenesis and the progression of liver cancer. Transforming growth factor-β (TGF-β) is a multifunctional cytokine that regulates cell growth, apoptosis, differentiation, cell motility, extracellular matrix production, and angiogenesis. TGF-β is upregulated in liver cancer tissue and elevated in the plasma of liver cancer patients [7,8], and is reported to activate multiple signaling pathways, including Smad-dependent and Smad-independent pathways involving liver cancer progression. While many reports have demonstrated that TGF-β activates Smad pathway mediating EMT, which contributes to hepatoma progression [9], it has been also demonstrated that the inhibition of p38MAPK, which is a downstream substrate of TGF-β-activating kinase 1 (TAK-1), reversed TGF-β-induced EMT [10]. Overall, developing agents to block TGF-β-mediated signal activation may provide a novel preventive and therapeutic strategy to suppress hepatoma progression.

**Figure 1:**
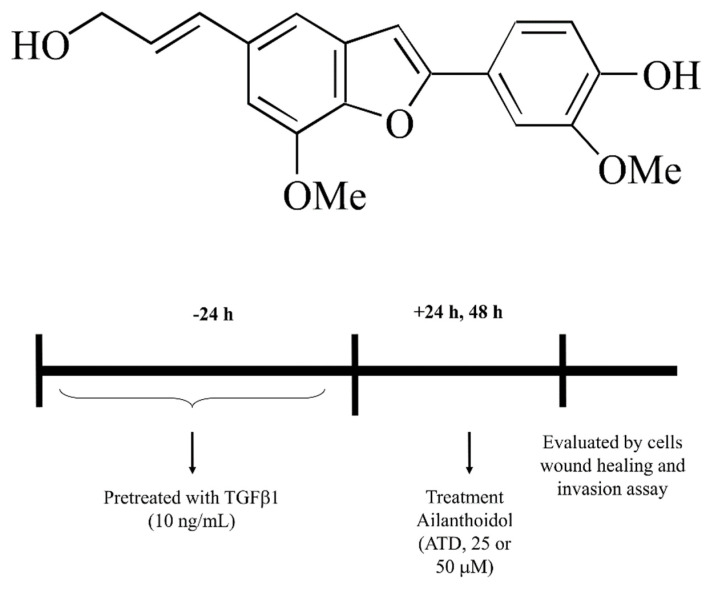
Chemical structure of ailanthoidol (ATD). Flow chart of ATD administered after initial of TGF-β1 pretreated HepG2.

Within phytochemicals, phenolic compounds called lignans have attracted the interest of medicinal and food chemists over years due to their broad range of biological activities including antioxidant, anti-inflammatory, antitumor, and antiviral [11,12,13]. Growth evidence demonstrate that lignan derivatives possess potential in disease prevention and therapy. Traditionally, lignans are classified into two types: classical lignans and neolignans. Classical lignans are phenylpropane (C6-C3) dimers linked in a β-β′ linkage and neolignans are composed of two C6-C3 units but not β-β′ linked [13]. Many classical lignans have been isolated from food such as sesame, whole-grain cereals, and legumes. Lignan-rich diets have been shown to protect against cardiovascular diseases and diabetes [14,15]. In addition, neolignans isolated from Magnolia bark, such as magnolol and honokiol, exhibit efficient effects on inhibiting or preventing the growth of various cancers [16]. Ailanthoidol (ATD) (Figure 1), a neolignan, has been isolated from the barks of *Zanthoxylum ailanthoidol* and demonstrated to possess antioxidant, anti-inflammatory, and anti-adipogenic activities [17,18,19]. Our previous study showed that ATD displayed antitumor promotion effect using the multistep skin cancer model induced by 12-o-tetradecanoylphobol-13-acetate [17]. While ATD is a neolignan, its anticancer properties have not been well clarified, thus, this study investigated the effect of ATD on TGF-β1-promoted HepG2 hepatoblastoma cell progression.

## 2. Materials and Methods

### 2.1. Materials

Dulbecco’s modified Eagle’s medium (DMEM), phosphate-buffered saline (PBS), fetal bovine serum (FBS, catalogue 10437028, Gibco BRL, Grand Island, NY, USA), penicillin/streptomycin/neomycin (PSN, catalogue 15240062, Gibco BRL, Grand Island, NY, USA), and trypsin-EDTA were purchased from Gibco Ltd. (Grand Island, NY, USA). TGF-β1 was from PeproTech, Rocky Hill, NJ, USA (Catalog Number: 100-21). Primary antibodies against integrin α3 (sc-374242), vimentin (sc-6260), N-cadherin (sc-8424), MMP2 (sc-13595), Smad 2/3 (sc-398844), and tubulin (sc-5286) were obtained from Santa Cruz Biotechnology, Inc. (Santa Cruz, CA, USA). Anti-p-p38MAPK (#9211), anti-p38MAPK (#9212), and anti-p-Smad 2/3 (#8828) were from Cell Signaling Technology (Beverly, MA, USA). ATD was provided by Dr. Lee and synthesized from 5-bromo-2-hydroxy-3-methoxybenzaldehyde, as previously reported [20]. Matrigel is a solubilized basement membrane preparation extracted from murine sarcomas, and contains proteoglycans, type IV collagen, laminin, and growth factors (Catalog Number: 356234 BD Biosciences, Bedford, MA, USA). Triton X-100, tris base, crystal violet, and all other materials were purchased from Sigma Chemical Co. (St. Louis, MO, USA).

### 2.2. Cell Culture

Human hepatoma HepG2 hepatoblastoma cells, obtained from the American Type Culture Collection (ATCC, Manassas, VA, USA), were cultured in Dulbecco’s modified Eagle’s medium (DMEM; Gibco BRL, Grand Island, NY, USA) supplemented with 10% fetal bovine serum (FBS) and 1% penicillin–streptomycin–neomycin. The cell line was maintained at 37 °C in a humidified incubator with 5% CO_2_. HepG2 is also referred to as a hepatoblastoma cell line (PubMed: 6248960 and 19751877). ATD was dissolved in DMSO (stock solution: 100 mM).

### 2.3. Cytotoxicity Assay

The HepG2 hepatoblastoma cells were seeded into 24-well culture plates (2 × 10^4^ cells/well), and then treated with various concentrations of ATD (0–100 μM) for 24 h and 48 h, respectively, at 37 °C in a humidified incubator with 5% CO_2_. After treatment, the medium was changed and incubated in the presence of 3-[4,5-dimethylthiazol-2-yl]-2,5-diphenyltetrazolium bromide (MTT) dye solution for 4 h. The number of viable cells was directly proportional to the production of formazan, which was then dissolved with dimethyl sulfoxide (DMSO) and the optical density was measured at 540 nm using an ELISA multiwall plate reader.

### 2.4. Wound Healing Assay

Cells (5 × 10^5^ cells per well) were cultured in 6-well plates and pretreated with or without (control group) TGF-β1 (10 ng/mL) for 24 h [21]. A wound was scratched by a 200 μL pipette tip. After washing with PBS, the cultured medium was replaced with fresh medium and cotreated with ATD (0, 10, 25, or 50 μM) and TGF-β1 (10 ng/mL) for an additional 24 h and 48 h, respectively, at 37 °C in a humidified incubator with 5% CO_2_. Cell migration into the wound area was photographed at 0, 24, and 48 h. The area of cell migration was evaluated in 6 random fields, compared with the wound area at 0 h using ImageJ software, and expressed as a percentage of the initial area. Data are represented as the mean ± SD of the three independent experiments.

### 2.5. In Vitro Cell Invasion Assay

The invasion experiment was assessed using transwell plates. Matrigel (BD Transduction Laboratories) was diluted at 1:25 with serum-free DMEM, and fifty microliters of diluted Matrigel was used to coat the upper insert membrane. Cells were pretreated with or without (control group) TGF-β1 (10 ng/mL) for 24 h, then co-treated ATD (0, 25, 50 μM) and TGF-β1 (10 ng/mL) for an additional 24 h at 37 °C in a humidified incubator with 5% CO_2_. Then the cells were harvested and the cells (2 × 10^4^ cells/well) in 200 μL of serum-free DMEM were added to the top chamber, while the complete growth medium (15% FBS) was placed in the lower chamber. After incubation for 24 h, the cells on the upper surface of the filter were wiped with a cotton swab, while the cells on the lower surface of the filters were fixed for 15 min with 4% paraformaldehyde and stained with 0.1% crystal violet for 20 min. Then, the cells that had invaded the lower surface of the filter were photographed by light microscopy. The invading cells in 6 randomly selected fields were determined and the counts were averaged.

### 2.6. Colony Assay

HepG2 hepatoblastoma cells (2 × 10^4^ cells/well) were seeded and incubated overnight. The cells were pretreated with or without (control group) TGF-β1 (10 ng/mL) for 24 h, then cotreated with ATD (0, 25, 50 μM) and TGF-β1 (10 ng/mL) for an additional 24 h at 37 °C in a humidified incubator with 5% CO_2_. Afterwards, the cells were fixed with 4% paraformaldehyde for 20 min, then stained with 0.1% crystal violet solutionin ethanol for 5 min and photographed. The colony number was quantified by ImageJ software that counts the spot of screan photos and expressed as a percentage of the control.

### 2.7. Preparation of Total Cell Extracts and Immunoblot Analysis

Cells were pretreated with or without (control group) TGF-β1 for 24 h, then co-treated with ATD (0, 25, 50 μM) and TGF-β1 (10 ng/mL) for an additional 24 h at 37 °C in a humidified incubator with 5% CO_2_. The cells were collected by trypsin-EDTA and lysed in a RIPA buffer (50 mM Tris-HCl, 150 mM NaCl, 1 mM EDTA, 1% NP40, and 0.5% deoxycholic acid containing PMSF, NaVO_4_, DTT, and protease inhibitor). After mixing for 30 min at 4 °C, the mixtures were centrifuged (10,000× *g*) for 10 min at 4 °C, and the supernatants were collected as whole-cell extracts. The protein content was determined with the Bio-Rad protein assay reagent using bovine serum albumin as the standard. After equal protein contents of the total cell extracts were boiled for 8 min, the extracts were separated by SDS-polyacrylamide gels and electrophoretically transferred to the PVDF membrane. Following SDS-polyacrylamide gel electrophoresis (PAGE) (10–12% running, 4% stacking), it was transferred to the PVDF member, and protein expression was detected. The blots were blocked with 5% skim milk in PBS for 1 h at room temperature, then incubated overnight with the indicated primary antibodies, followed by incubation with horseradish peroxidase-conjugated goat anti-mouse (or rabbit) IgG for 1 h. Proteins were then blotted onto NC membranes, and reacted with the primary antibodies (integrin α3 (1:10,000), vimentin (1:10,000), N-cadherin (1:2000), MMP2 (1:2000), Smad 2/3 (1:2000), and tubulin (1:10,000) were obtained from Santa Cruz Biotechnology, Inc. (Santa Cruz, CA, USA). Anti-p-p38MAPK (1:1000), anti-p38MAPK (1:1000), and anti-p-Smad 2/3 (1:2000) were from Cell Signaling Technology (Beverly, MA, USA)). The secondary antibody was a peroxidase-conjugated goat anti-mouse antibody (1:10,000), obtained from Santa Cruz Biotechnology, Inc., CA, USA. After binding for 1 h, the immune-reactive bands were revealed by enhanced chemiluminescence using an ECL commercial kit. The relative image density was quantitated by densitometry.

### 2.8. Statistical Analysis

Data were expressed as mean ± S.D. (n = 6/group) of three independent experiments and analyzed by one-way ANOVA with Tukey’s Multiple Comparison Test. Significant differences were established at *p* *<* 0.05. The data were analysed using the SAS software statistical package SigmaPlot, version 9.0 (SAS Institute Inc., Cary, NC, USA).

## 3. Results

### 3.1. The Cytotoxicity of ATD on HepG2 Hepatoblastoma Cells

**Figure 2:**
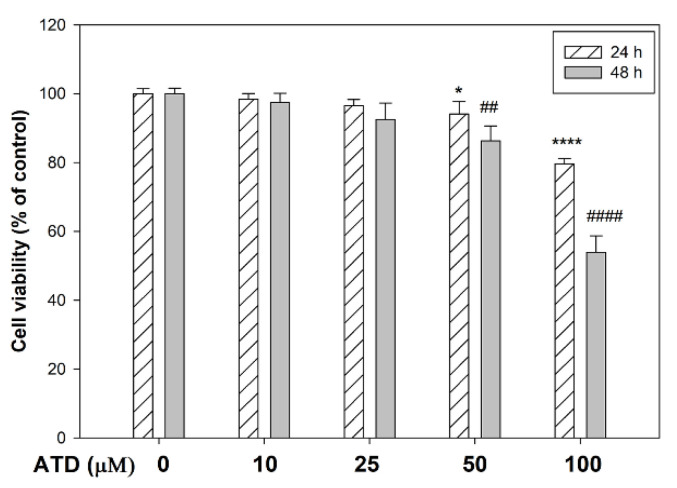
The cytotoxic effect of ATD on the HepG2 hepatoblastoma cells was measured by MTT assay. Cells were seeded in 24-well plates at 2 × 10^4^ cells/well. After attachment, cells were treated with various concentrations of ATD (0–100 μM) for 24 h and 48 h. The cell viability was determined by MTT assay as described in the text. Data are represented as the mean ± SD from three independent experiments. * *p* < 0.05, **** *p* < 0.0001, compared with the control group (0.2% DMSO) of treatment for 24 h. ## *p* < 0.01, #### *p* < 0.0001, compared with the control group (0.2% DMSO) of treatment for 48 h.

The cytotoxic effect of ATD was evaluated in HepG2 hepatoblastoma cells by MTT assay. Various concentrations of ATD (0–100 μM) were applied to the HepG2 hepatoblastoma cells for 24 and 48 h. As shown in Figure 2, doses of ATD up to 25 μM exhibited no significant growth inhibition on HepG2 cells regandless of treatment for 24 h or 48 h. In addition, 50 μM ATD treated for 24 h inhibited cell viability as compared to control group (*p* < 0.05), but that was not significant as compared to the 25 μM ATD treated group. IC50 of ATD on HepG2 cells for 48 h was near 100 μM. To clarify the role of ATD on TGF-β1-induced progression of liver cancer, 0–50 μM concentrations of ATD were used in this study.

### 3.2. Inhibitory Effect of ATD on TGF-β1-Promoted Migration and Invasion of HepG2 Hepatoblastoma Cells

**Figure 3:**
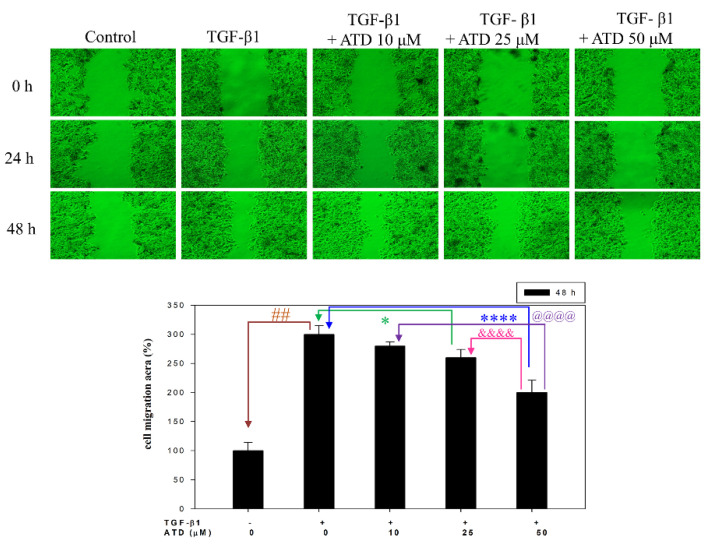
Effect of ATD on TGF-β1-induced migration of HepG2 hepatoblastoma cells by wound-healing assay. Cells were cultured in 6-well plates and pretreated with or without (control group) 10 ng/mL of TGF-β1 for 24 h. A wound was scratched by a 200 μL pipette tip. After washing with PBS, cultured medium was replaced with fresh medium and with or without coadministration of TGF-β1 and ATD (0, 10, 25, or 50 μM) for 24 h and 48 h, then photographed at 0 h, 24 h, and 48 h. Migration activity of cotreated with TGF-β1 and ATD for 48 h was quantified with ImageJ software. Data are represented as the mean ± SD from three independent experiments. ##: TGF-β1 vs. control, *p* < 0.01; *: TGF-β1 vs. TGF-β1+ 25 μM ATD, *p* < 0.05; ****: TGF-β1 vs. TGF-β1+ 50 μM ATD, *p* < 0.0001; &&&&: TGF-β1+ 25 μM ATD vs. TGF-β1+ 50 μM ATD, *p* < 0.0001; @@@@: TGF-β1+ 10 μM ATD vs. TGF-β1+ 50 μM ATD, *p* < 0.0001.

**Figure 4:**
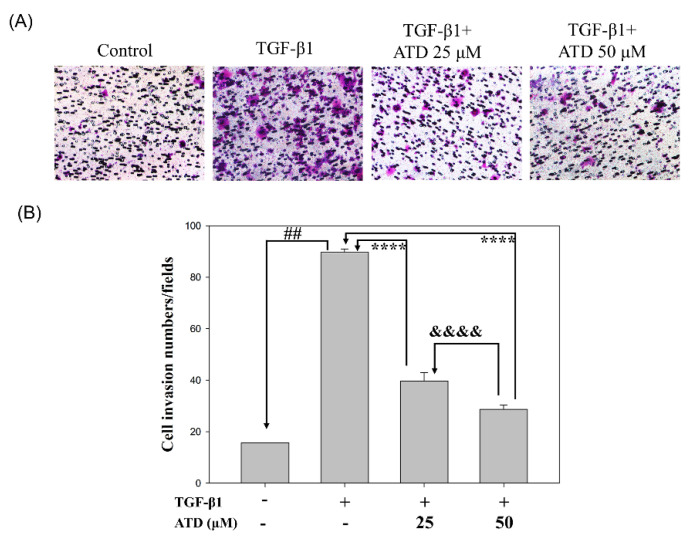
Effect of ATD on invasion induced by TGF-β1 in HepG2 hepatoblastoma cells. Cells were pretreated with or without (control group) TGF-β1 for 24 h, then co-treated indicated concentration of ATD and TGF-β1 for an additional 24 h. Cells were harvested and seeded onto upper chamber which was coated with Matrigel. The transwell chamber assay was performed as described in the text. (**A**) After incubation for 24 h, invasion cells in the lower surface of membrane were fixed, then stained with crystal violet solution and photographed at 200× magnification. (**B**) The invasion cells were quantified in 6 randomly selected fields per well (n = 6) using a light microscope. Data are represented as the mean ± SD. ##: TGF-β1 vs. control, *p* < 0.01; ****: TGF-β1 vs. TGF-β1+ 25 μM ATD or + 50 μM ATD, *p* < 0.0001; &&&&: TGF-β1+ 25 μM ATD vs. TGF-β1+ 50 μM ATD, *p* < 0.0001.

Since metastasis depends on increased motility or invasion of tumor cells to successfully colonize a secondary site, the effect of ATD on TGF-β1-promoted cell motility growth in HepG2 hepatoblastoma cells was evaluated by wound healing assay. According to photographs (Figure 3), TGF-β1 treated for 48 h after the created wound displayed obvious migration growth. The continuous rapid movement was observed for HepG2 cells, but a resultant movement of a cell migration front was clearly evident at 48 h, where a highly confluent monolayer region gradually migrated into the cell-free ‘scratch’ region. In the presence of TGF-β1, migration was significantly increased after 24 and 48 h, whereas treatment with ATD lead to an inhibition of cell migration in a dose-dependent manner, shown as 91%, 83%, and 72% (Figure 3). As shown in Figure 3, TGF-β1 significantly (*p* < 0.01) promoted cell migration, which was inhibited when cotreated with ATD (25 μM and 50 μM) for 48 h in HepG2 hepatoblastoma cells significantly. We further examined whether ATD affected TGF-β1-promoted invasion in HepG2 hepatoblastoma cells using the transwell assay, and the results show that treatment with ATD (25 μM and 50 μM) significantly inhibited TGF-β1-promoted cell invasion (Figure 4).

### 3.3. Effect of ATD on Colonization of HepG2 Hepatoblastoma Cells

**Figure 5:**
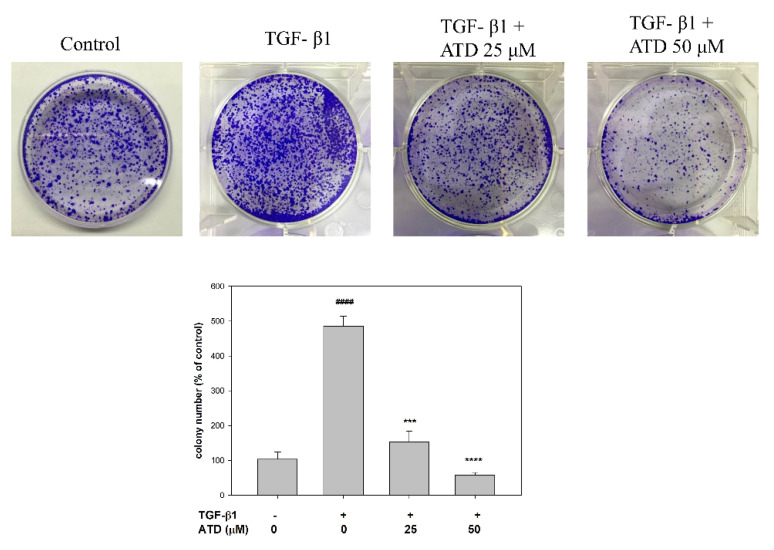
Effect of ATD on TGF-β1-promted growth in HepG2 hepatoblastoma cells by colony formation assay. After treatment with ATD or cotreatment ATD and TGF-β1 for 24 h, 1000 cells were seeded on 6-well plate and cultured for 2 weeks, then cells were fixed with methanol and stained with crystal violet. Colony numbers were quantified by ImageJ software. Data are represented as the mean ± SD from the three independent experiments. # *p* < 0.05, ## *p* < 0.01, #### *p* < 0.0001, compared with the control group treated (0.2% DMSO). ***: TGF-β1 vs. TGF-β1+ 25 μM ATD, *p* < 0.001; ****: TGF-β1 vs. TGF-β1+ 50 μM ATD, *p* < 0.0001.

To further evaluate the effect of ATD on the TGF-β1-promoted colonization growth in vitro, a colony assay was performed. After various administrations of TGF-β1 and ATD for 24 h, one thousand cells were counted and seeded onto a dish, and the culture was maintained for 2 weeks. The result showed that administration of TGF-β1 increased the colony formation of HepG2 cells as compared to the control group (Figure 5). The cotreatment group showed that ATD significantly reduced TGF-β1-promoted colony formation of HepG2 cells (Figure 5).

### 3.4. Effect of ATD on TGF-β1-Induced Cell Scattering

**Figure 6:**
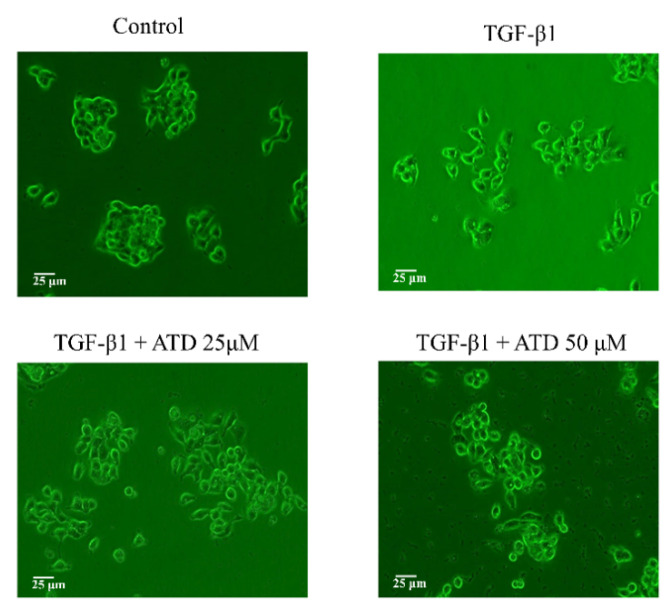
Reverse effect of ATD on TGF-β1-induced cell scattering in HepG2 hepatoblastoma cells. The cell was applied with or without (control group) TGF-β1 for 24 h, and then TGF-β1 co-treated with or without ATD for an additional 24 h. The cell was observed under phase contrast microscopy (200×) and photographed.

Cell scattering is a critical manifest of several physiological and pathological processes such as tissue regeneration and cancer cell invasion. In the morphology observation, we found that the cells exhibited remarkable cell scattering after treatment with TGF-β1, while the cultured HepG2 hepatoblastoma cells displayed a tight junction. Figure 6 shows that treatment with TGF-β1-induced cell scattering was reversed by co-treatment with ATD.

### 3.5. Alterations of ATD on TGF-β1-Induced Progression Associated Protein Marker Expression in HepG2 Hepatoblastoma Cells

**Figure 7:**
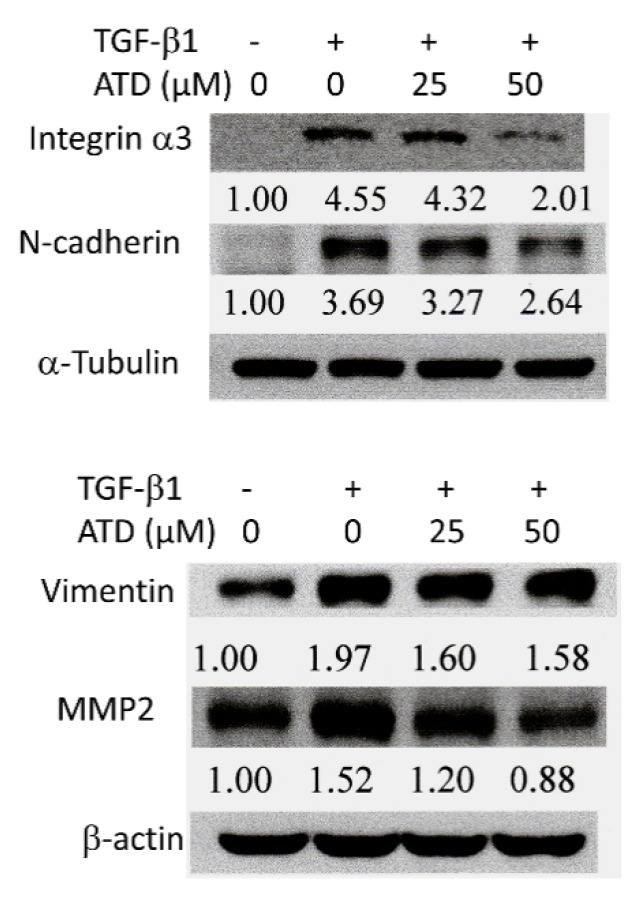
Effect of ATD on the cancer progression markers induced by TGF-β1 in HepG2 hepatoblastoma cells. Following the TGF-β1 and ATD treatment as described in the text, the cell lysates were prepared and subjected to the immunoblotting analysis using antibodies against integrin-α3, vimentin, N-cadherin, MMP2, and α-tubulin or β-actin as loading control. The relative image density was quantitated by densitometer.

Due to their diverse roles, integrins are key players in cancer metastasis, such as in cell motility [22]. It has been reported that TGF-β1 plays a critical role in liver cancer progression by stimulating integrin α3 expression [23]. In addition, the expression of N-cadherin and vimentin facilitates the process of EMT and promotes migration and invasion. ECM degradation by MMPs also promotes cell migration and invasion. This study examined the effect of ATD on TGF-β1-induced expression of cancer cell progression associated proteins, and the results showed TGF-β1 promoted the expression of integrin α3, N-cadherin, vimentin, and MMP2, while TGF-β1 cotreated with ATD inhibited such expression (Figure 7). It implicated that ATD suppressed TGF-β1-induced expression of progression-associated proteins.

### 3.6. ATD Inactivating TGF-β1-Induced p38MAPK and SMAD Signaling

**Figure 8:**
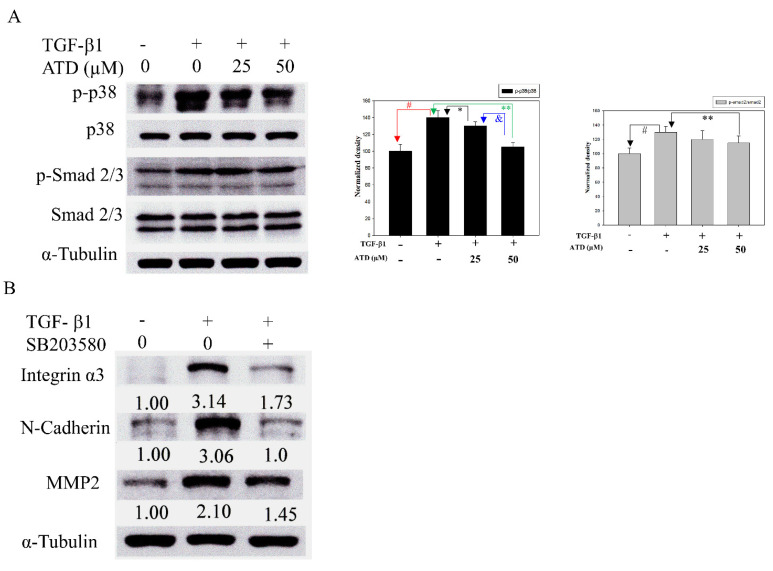
Effect of ATD on TGF-β1-induced p38MAPK and Smad 2/3 pathway. (**A**) After treatment of TGF-β1 and ATD as described in the text, the cell lysates were prepared and subjected to immunoblotting analysis using antibodies against p-p38MAPK, p-Smad 2/3, p38MAPK, Smad 2/3, and α-tubulin (loading control). The average densitometric value was shown as mean ± SD (*n* = 3). #: TGF-β1 vs. control, *p* < 0.05; *: TGF-β1 vs. TGF-β1+ 25 μM ATD, *p* < 0.05; ** TGF-β1 vs. TGF-β1+ 50 μM ATD, *p* < 0.01; &: TGF-β1+ 25 μM ATD vs. TGF-β1+ 50 μM ATD, *p* < 0.05. (**B**) Cells were pretreated with or without TGF-β1 for 24 h, then co-treated with 5 μM p38 MAPK inhibitor (SB203580) and TGF-β1 for an additional 24 h. The cell lysates were subjected to immunoblotting analysis using antibodies against integrin-α3, N-cadherin, MMP2, and α-tubulin (loading control). The relative image density was quantitated by a densitometer.

In addition to activating the Smad signaling pathway, TGF-β1 activates kinase-1 (TAK1), which in turn triggers the activation of downstream p38MAPK [24]. p38MAPK has been implicated in a wide range of complex biologic processes, such as migration and invasion in solid tumors [25]. In order to resolve the mechanisms by which ATD inhibited migration and invasion, as induced by TGF-β1 in HepG2 hepatoblastoma cells, we examined the phosphorylation of p38MAPK and Smads. While TGF-β1 upregulated phosphorylation of Smad2 and p38MAPK, ATD (25 or 50 μM) co-treated significantly inhibited the phosphorylation of p38MAPK, and ATD (50 μM) co-treated significantly inhibited the phosphorylation of Smad2 (Figure 8A). In addition, SB203580, which is a p38MAPK inhibitor, was used to confirm the effect of blocking p38MAPK and alleviating the TGF-β-induced progression associated protein expression. The results showed that SB203580 (5 μM) inhibited TGF-β-enhanced expression of integrin α3, N-cadherin, and MMP2 (Figure 8B).

## 4. Discussion

Most liver cancer occurs in patients with chronic liver disease, which appears as hepatitis, fibrosis, and cirrhosis, and suggests that liver cancer is an inflammation-associated cancer [26]. Cytokines are key mediators of inflammation. TGF-β is a multifunctional cytokine, which plays a critical role in arranging a favorable microenvironment for tumor growth and progression. TGF-β promotes hepatocellular carcinoma progression via an autocrine or paracrine growth factor, which induces microenvironment changes that trigger cell migration and invasion [27]. TGF-β1 has been reported to promote HepG2 hepatoblastoma cell migration and invasion [28]. The occurrence of EMT is affected by various factors such as TGF-β1 [29]. Furthermore, TGF-β1 stimulates integrin α3 expression, which has been suggested to play an important role in liver cancer invasiveness [23]. In the present study, in addition to reversing TGF-β1-promoted cell scattering, ATD attenuated TGF-β1-induced the expression of cancer progression-associated proteins (Figure 7), which is a novel action of ATD on antitumor progression.

**Figure 9:**
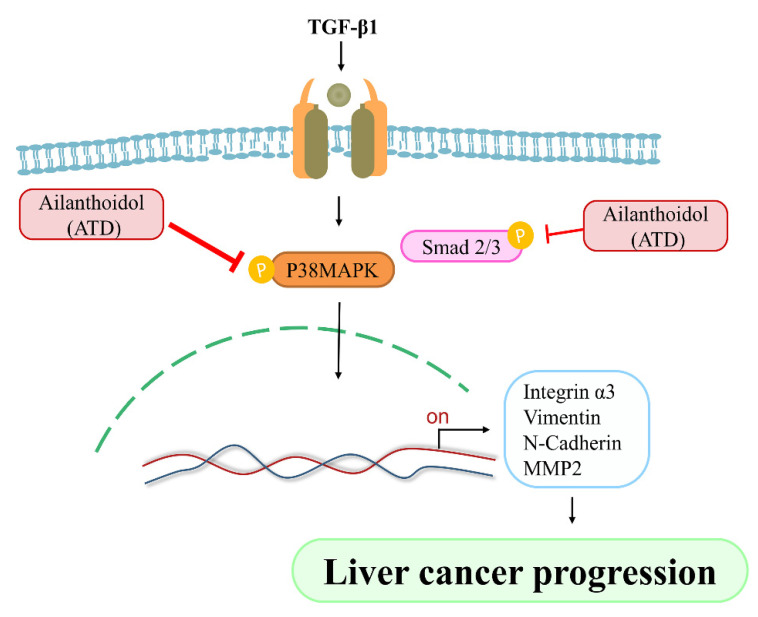
Effect of anti-tumor potential of Ailanthoidol (ATD), ATD blocks TGF-β1 activated two signaling axes: P38MARK pathway and Smad 2/3 pathway, resulting in down-regulation of liver cancer progression associated proteins.

Cancer is a multiple-cause disease in which many signaling pathways are affected [30]. MAPK mediates a wide variety of cellular behaviors in response to extracellular stimuli. As one of the main subgroups, p38MAPK has been implied in a broad range of complicated biological processes, including cell proliferation, cell death, cell differentiation, cell migration, and cell invasion. The specific function of p38MAPK appears to depend not only on the cell type, but also on the stimulants. Liver cancer development is controlled by both extracellular factors and intracellular signaling pathways. TGF-β1, which is a cytokine, plays a critical role in liver cancer progression involving Smad-dependent and independent pathways [31]. TGF-β1 activates the TGF-βI/II receptor, which phosporylates Smad2 and Smad3, leading to the formation of a heteromeric Smad complex with cytosolic Smad4. The Smad complex translocates to the nucleus, where it regulates gene transcription of several transcription factors involved in the EMT induction. In addition to the Smad pathway, the activation of the p38MAPK pathway has been reported to contribute to the EMT in the primary tumor, as well as to the acquisition of tumor cell migration and invasion. It has been reported that TGF-β1 activates Smad and TAK1/p38MAPK pathways targeting ATF-2 synergistically induce trans-activation capacity [32]. Silencing ATF2 demonstrates anticancer effects in liver cancer [33]. This study found that ATD could attenuate TGF-β1-induced phosphorylation of nuclear ATF2 (data not shown), thus, whether ATD inactivated other transcription factors in the downstream of p38MAPK requires further clarification. The results show that ATD attenuated TGF-β1-promoted HepG2 hepatoblastoma cell progression by inhibiting Smad-dependent and independent pathways (Figure 9). According to Figure 8, it also reveals that ATD reduced TGF-β1-induced expression of invasion-associated proteins in large part through blocking p38MAPK signaling.

Plants are the major food and pharmaceutical origins of humans. Some phytochemicals, such as alkaloids, diterpenoids, and sesquineterpenes, display therapeutic potential in cancer [34]. However, these therapeutic phytochemicals are also associated with adverse side effects, such as cardiovascular diseases, vomiting, renal dysfunction, and myelotoxicity. In addition, certain polyphenol-rich foods have been reported to reduce the incidence and mortality rates of cancer, as well as delay cancer progression [35]. However, past studies have found that in order to exert the health benefits, the required dose of polyphenols is higher than humans can consume [36]. Therefore, it is imperative to develop chemotherapy or chemoprevention substances with minimal side effects and good bioavailability. Thus, it has been suggested that plants are still a reservoir possessing enormous potential to provide chemotherapy or chemoprevention. Recently, neolignan derivatives are suggested to possess great potential for chemoprevention and treatment [37]. In addition, it has been reported that compounds containing heterocyclic moieties can boost salt formation properties, which are important for bioavailability [38]. Recently, neolignan derivatives were suggested to possess great potential for chemoprevention and treatment [16]. Several neolignans from the Magnolia family are biphenyl neolignans found to possess a good safety prolife while ATD is a neolignan with a 2-arylbenzofuran skeleton. [16] In addition, it has been reported that compounds containing heterocyclic moieties can boost salt formation properties, which are important for bioavailability [38]. For real application, animal studies of ATD are required to clarify the in vivo properties such as adsorption, distribution, metabolism, and elimination in the future. In addition, growing evidence has been shown that chronic inflammation is associated with high incidence of several cancers [6]. Targeting inflammatory pathways has proven effective in preventing the formation of colonic tumors and their malignant progression in both preclinical and clinical studies. Synthetic non-steroidal anti-inflammatory drugs have been recognized as potential colorectal cancer chemopreventive agents; however, most of these synthetic agents are associated with sometimes fatal or unwanted side effects [39]. A large amount of evidence supports the effectiveness of natural phytochemicals with anti-inflammatory activity in the prevention and treatment of cancer [40]. Our previous study found that ATD inhibited 12-o-tetradecanoylphobol-13-acetate induced COX-2 expression and myeloperoxidase activation. It implicated that ATD possessed anti-inflammatory bioactivity [17]. Kim and Jun reported that ATD suppresses lipopolysaccharide-stimulated inflammatory in RAW 264.3 cells and endotoxin shock in mice [18]. The present investigation demonstrates that ATD exerts antitumor progression properties by modulating the effect of cytokine. While ATD is a neolignan with heterocyclic moiety and possesses antiinflammatory activity, whether it can play a role in chemoprevention requires further investigation.

## 5. Conclusions

This study first demonstrated a novel mechanism—that ATD exerted antitumor potential through suppressing TGF-β1-promoted HepG2 hepatoblastoma cell progression, which involves blocking p38MAPK and Smad 2 signaling (Figure 9). Further investigation is required to elucidate the universal anticancer properties of ATD against human hepatoblastoma cell lines.

## Data Availability

All relevant data are within the paper.
